# Digital quantitation of bridging fibrosis and septa reveals changes in natural history and treatment not seen with conventional histology

**DOI:** 10.1111/liv.16092

**Published:** 2024-09-09

**Authors:** Nikolai V. Naoumov, David E. Kleiner, Elaine Chng, Dominique Brees, Chandra Saravanan, Yayun Ren, Dean Tai, Arun J. Sanyal

**Affiliations:** ^1^ Division of Medicine University College London London UK; ^2^ Laboratory of Pathology, Post‐Mortem Section National Cancer Institute Bethesda Maryland USA; ^3^ Histoindex Pte. Ltd. Singapore Singapore; ^4^ Novartis Pharma AG Basel Switzerland; ^5^ Novartis Institute of Biomedical Research Cambridge Massachusetts USA; ^6^ Stravitz‐Sanyal Institute of Liver Disease and Metabolic Health Virginia Commonwealth University School of Medicine Richmond Virginia USA

**Keywords:** digital pathology with artificial intelligence, liver fibrosis, metabolic dysfunction‐associated steatohepatitis, quantitative assessment fibrosis regression, second harmonic generation microscopy

## Abstract

**Background and Aims:**

Metabolic dysfunction‐associated steatohepatitis (MASH) with bridging fibrosis is a critical stage in the evolution of fatty liver disease. Second harmonic generation/two‐photon excitation fluorescence (SHG/TPEF) microscopy with artificial intelligence (AI) provides sensitive and reproducible quantitation of liver fibrosis. This methodology was applied to gain an in‐depth understanding of intra‐stage fibrosis changes and septa analyses in a homogenous, well‐characterised group with MASH F3 fibrosis.

**Methods:**

Paired liver biopsies (baseline [BL] and end of treatment [EOT]) of 57 patients (placebo, *n* = 17 and tropifexor *n* = 40), with F3 fibrosis stage at BL according to the clinical research network (CRN) scoring, were included. Unstained sections were examined using SHG/TPEF microscopy with AI. Changes in liver fibrosis overall and in five areas of liver lobules were quantitatively assessed by qFibrosis. Progressive, regressive septa, and 12 septa parameters were quantitatively analysed.

**Results:**

qFibrosis demonstrated fibrosis progression or regression in 14/17 (82%) patients receiving placebo, while the CRN scoring categorised 11/17 (65%) as ‘no change’. Radar maps with qFibrosis readouts visualised quantitative fibrosis dynamics in different areas of liver lobules even in cases categorised as ‘No Change’. Measurement of septa parameters objectively differentiated regressive and progressive septa (*p* < .001). Quantitative changes in individual septa parameters (BL to EOT) were observed both in the ‘no change’ and the ‘regression’ subgroups, as defined by the CRN scoring.

**Conclusion:**

SHG/TPEF microscopy with AI provides greater granularity and precision in assessing fibrosis dynamics in patients with bridging fibrosis, thus advancing knowledge development of fibrosis evolution in natural history and in clinical trials.

**Abbreviations**
AI: artificial intelligence
BL: baseline
EOT: end of treatment
FXR: farnesoid X receptor
H&E: haematoxylin and eosin
MASH: metabolic dysfunction‐associated steatohepatitis
MASLD: metabolic dysfunction‐associated steatotic liver disease
MT: Masson Trichrome
NAFLD: non‐alcoholic fatty liver disease
NASH: non‐alcoholic steatohepatitis
NASH CRN: NASH Clinical Research Network
P/N/R analysis: grouping patients according to fibrosis dynamics as Progressive, No‐change, or Regressive
SHG/TPEF: second harmonic generation/two‐photon excitation fluorescence microscopy
TXR: tropifexor

**Key points**

- Metabolic dysfunction‐associated steatohepatitis (MASH) is a condition where fat accumulates in the liver cells due to metabolic problems, which leads to tissue damage, inflammation, and a scar‐like formation called fibrosis.
- We used second harmonic generation/two‐photon excitation fluorescence (SHG/TPFE) microscopy and artificial intelligence (AI), a more sensitive and standardised methodology, to look closely at changes in fibrosis in patients with MASH and to better assess the treatment response.
- The present study demonstrates that the use of SHG/TPEF microscopy combined with AI provides information with greater precision and granularity that allows assessment of changes in fibrosis in patients with MASH and liver fibrosis with greater detail and reproducibility.

## 1 INTRODUCTION

Nonalcoholic steatohepatitis (NASH) is a progressive form of non‐alcoholic fatty liver disease (NAFLD), the most common liver disease worldwide, which is associated with hepatocyte damage driven by toxic lipids, chronic inflammation, and varying degrees of liver fibrosis.^1^, ^2^, ^3^ Recently, the NAFLD Nomenclature Consensus Group involving international experts from 56 countries determined a new terminology, where metabolic dysfunction‐associated steatotic liver disease (MASLD) was chosen to replace NAFLD, and metabolic dysfunction‐associated steatohepatitis (MASH) to replace NASH.^4^

Earlier retrospective studies, as well as recent prospective studies, have all shown that the degree of liver fibrosis is the principal feature that predicts the clinical outcomes and mortality in patients with MASLD.^5^, ^6^, ^7^, ^8^, ^9^, ^10^ The growing prevalence of MASH globally, with related morbidity and mortality has focused the coordinated efforts of clinical investigators, regulatory authorities and pharmaceutical companies to address this unmet need. The FDA views MASH with liver fibrosis as a serious, life‐threatening condition and an important area of investigational drug development.^11^ Assessment of liver histology is essential for defining patients' eligibility and the primary efficacy endpoints in Phase 2b and Phase 3 clinical trials.

MASH with bridging fibrosis (fibrosis stage F3) is a critical stage in the evolution of the disease, which has the highest incidence of liver‐related events and all‐cause mortality in the pre‐cirrhotic MASLD group.^9^ Furthermore, up to 22% of patients with bridging fibrosis were shown to progress to MASH cirrhosis over a median follow‐up of 2 years.^7^, ^8^, ^12^ Importantly, in a recent analysis of liver biopsies from patients with pre‐cirrhotic MASH, we have shown that liver fibrosis F3 stage is very dynamic with marked fibrosis regression, in a proportion of patients, which was more pronounced than in patients with F2 fibrosis stage.^13^ However, the F3 stage is a broad category and despite a marked reduction in the amount of collagen deposition, the presence of a delicate fibrous bridge would still be considered as stage 3 disease.^14^, ^15^, ^16^ All grading and staging systems used in chronic liver disease were developed based on histological changes in untreated individuals, and they do not necessarily account for changes occurring after successful therapy, especially for fibrosis regression, which is poorly assessed by existing staging systems.^17^ This inability to reflect intra‐stage changes is a major limitation of the current scoring systems and poses a challenge for accurate assessment of the progression or regression of bridging fibrosis in the natural history or in clinical trials, which is increasingly recognised.^18^, ^19^

An in‐depth analysis of treatment‐induced changes of bridging septa in patients with chronic hepatitis B suggested that in addition to the conventional evaluation of liver fibrosis stage, there is utility in the assessment of the balance between progressive and regressive features defining three categories of fibrosis: predominantly progressive, predominately regressive, and indeterminate.^20^ The main advantage of the proposed ‘*Beijing classification*’ for bridging fibrosis in chronic hepatitis B is that it includes not only the extent (stage) of fibrosis but also additional characterisation indicating the direction of fibrosis evolution, namely if the specimen shows predominantly regressive, progressive, or indeterminate features.

Recent developments in second harmonic generation/two‐photon excitation fluorescence (SHG/TPEF) microscopy, which uses unstained, formalin‐fixed tissue sections uncovered new insights in fibrosis dynamics with sensitive, precise, and reproducible digital evaluation of liver fibrosis. The amount, topography, and architecture of collagen fibres in the liver biopsy are determined by quantitative assessment of individual collagen features as qFibrosis—a cumulative index based on measuring more than 100 collagen parameters on a continuous scale, for example collagen fibre length, width, area and fibre intersections.^21^, ^22^, ^23^, ^24^ The application of SHG/TPEF methodology with computer‐assisted analyses in chronic hepatitis B and MASH patients with bridging fibrosis has revealed marked intra‐stage changes with fibrosis progression or regression.^13^, ^25^ Using this methodology, the current study undertook an in‐depth, quantitative evaluation of changes in liver fibrosis overall and individual septa parameters in a homogenous, well‐characterised group of patients with bridging MASH fibrosis (F3 stage), who participated in the FLIGHT‐farnesoid X receptor (FXR) clinical trial (NCT02855164). The objectives of this analysis were (1) to quantitatively assess and graphically present intra‐stage changes of liver fibrosis from baseline (BL) to end of treatment (EOT); (2) to compare progressive and regressive types of fibrous septa and quantitatively assess the changes in individual septa parameters from BL to EOT and (3) to evaluate whether the BL value of qFibrosis as a continuous readout, or individual septa parameters can predict treatment response with liver fibrosis regression versus non‐regression in patients with MASH F3 fibrosis.

## 2 PATIENTS AND METHODS

### 2.1 Study population

This is a post hoc analysis of liver fibrosis dynamics in a homogenous group of 57 patients with biopsy‐proven MASH, all with bridging fibrosis (F3 stage) according to the clinical research network (CRN) scoring system at BL, who participated in the FLIGHT‐FXR clinical trial (NCT02855164). Briefly, FLIGHT‐FXR is a phase II randomised, double‐blind, placebo‐controlled, dose‐finding study with an adaptive design consisting of 3 sequential Parts (A, B, and C) assessing the safety and efficacy of tropifexor (TXR), a non‐bile acid FXR agonist, in patients with MASH. The study design and all safety and efficacy findings from this trial were published in detail.^26^ All 57 patients with F3 fibrosis stage who were enrolled in Part C of the FLIGHT FXR study were included in the present analysis. According to the trial protocol, these patients completed 48 weeks of treatment with a placebo (*n* = 17) or two doses of TXR—140 μg (*n* = 22) or 200 μg (*n* = 18), and underwent a liver biopsy at EOT.

### 2.2 Conventional liver histology

BL and EOT liver biopsies were formalin‐fixed, embedded in paraffin and 5 μm sections were stained using haematoxylin and eosin (H&E) and Masson trichrome (MT) stains. Stained liver sections were evaluated by the study central histopathologist to confirm eligibility before randomisation. After all patients completed their participation in the trial, BL and EOT (week 48) biopsies of each patient were assessed at the same time by the central histopathologist, who was blinded to participant identification, treatment, and temporal sequence of the samples. The MASH features and fibrosis stage were scored using the semiquantitative NASH CRN scoring system.^15^, ^26^

### 2.3 SHG/TPEF microscopy and AI algorithms

Unstained sections from 114 paired liver biopsies (BL and EOT) from 57 patients with NASH CRN F3 fibrosis were examined using SHG/TPEF microscopy. The liver sections were de‐paraffinised, followed by tissue scanning on Genesis® 200 (a fully automated, stain‐free two‐photon fluorescence imaging microscope) and analysed using artificial intelligence (AI)‐based algorithms (HistoIndex Pte. Ltd), as described previously.^13^, ^27^, ^28^ Samples were laser‐excited at 780 nm, SHG signals were recorded at 390 nm, and TPEF signals were recorded at 550 nm. Images were acquired at 20× magnification with a 512 × 512 pixels resolution; each image tile had a dimension of 200 × 200 μm. Multiple adjacent image tiles were captured to encompass the whole tissue area in each slide. The liver specimens included in this study were large cores of liver tissue and the AI computer‐assisted measurement of 114 biopsies showed a median length of 36.1 mm (range from 12.9 to 71.7 mm), with a median number of portal tracts of 40 (range from 6 to 119). Thus, the liver specimens used in this study were well above the minimum sampling size required for qFibrosis analyses, as defined in a recent study.^29^ The SHG/TPEF examination and analyses were performed blinded to type of treatment, timepoint, central pathologist's scoring, and without knowledge of any results from clinical trial investigations.^13^

### 2.4 Quantitative analyses of liver fibrosis overall

Similar to the approach used in a recent study of fibrosis changes in a mouse model of MASH, the qFibrosis index, reflecting the severity of liver fibrosis in the liver specimen overall was determined by measuring 184 collagen features on a continuous scale.^30^ Fifteen key collagen features were selected for in‐depth analyses (Figure S2A S1). qFibrosis calculation is based on normalised collagen parameters expressed as the number of units per μm^2^ and provides an unbiased, highly reproducible assessment of the severity of liver fibrosis.^27^, ^30^ In addition, the proportion of fibrosis area as a percentage of the total fibrosis area was determined in 5 operator defined regions of the liver lobules. These regions included: (1) Portal fibrosis, collagen fibrils located within the portal tract; (2) Peri‐Portal fibrosis, collagen within 100 μm circumferentially around the portal tract; (3) Zone 2 Perisinusoidal fibrosis, collagen depositions located within the area between Zone 1 and Zone 3 of liver lobule; (4) Peri‐Central Fibrosis, collagen fibrils located within 100 μm circumferentially around the central vein and (5) Bridging fibrosis, collagen deposition within bridging septa.^13^

Radar maps were developed as a novel approach for visualising fibrosis changes in liver lobules. The changes in fibrosis (as determined by the mean percentage change in fibrosis area overall and in different regions), from BL to EOT were presented in a map with 5 different dimensions according to the regions measured.

Considering that MASH treatment with compounds, which markedly reduce liver fat content, can alter the area used for fibrosis quantitation, especially when comparing pre‐ and post‐treatment fibrosis, a steatosis correction was applied when assessing fibrosis dynamics within the liver lobule, as described previously.^13^

### 2.5 Quantitative analyses of individual septa parameters

Based on the analyses of SHG/TPEF images, the fibrous septa in liver specimens were categorised as progressive, regressive, or intermediate septa according to the classification previously reported in patients with chronic hepatitis B.^20^, ^31^ *Progressive septa* are broad, mostly (more than 50%) with loosely aggregated collagen fibres, and irregular borders invading liver parenchyma, with moderate to marked cellular content (Figure S2A). *Regressive septa* are mostly (more than 50%) thin with densely compacted stroma, largely acellular, having sharp borders with liver parenchyma, and some septa have broken collagen fibres. *Indeterminate septa*: defined as an uncertain mix/balance between progressive and regressive features.

AI‐based software measured 12 individual septa parameters representing the septa area, septa length, septa width, number, and characteristics of collagen fibres within septa, etc. The average area of all septa in each liver specimen was measured in BL and EOT liver biopsies and expressed as a median area and range for each liver biopsy as a new cumulative parameter for assessing the dynamics of septal fibrosis (Figure S2B,C).

### 2.6 Statistical analyses

The quantitative readouts of fibrosis dynamics from BL to EOT were defined as fibrosis progression or fibrosis regression by a 10% or more relative increase or decrease in the value of respective parameters. We have previously demonstrated only a 5% variance of SHG repeated measures of qFibrosis.^30^ Based on this, we assigned a 10% or greater change in qFibrosis measures to be a significant change, similar to a recent study of fibrosis progression or regression in a mouse model of MASH.^30^

Fibrosis changes (BL to EOT) between the placebo and TXR treatments were compared using Progressive/No Change/Regressive (P/N/R) analysis. The number of cases in each subgroup was divided by the total number of patients in each arm to obtain the percentages shown. A Chi‐square test was applied to the grouping patients according to fibrosis dynamics as Progressive, No‐change, or Regressive (P/N/R analysis). The average septa area per liver specimen was calculated and the changes were compared using the Wilcoxon rank sum test.

## 3 RESULTS

### 3.1 Comparison of digital fibrosis quantitation versus conventional fibrosis staging in untreated patients with F3 MASH biopsies

**FIGURE 1:**
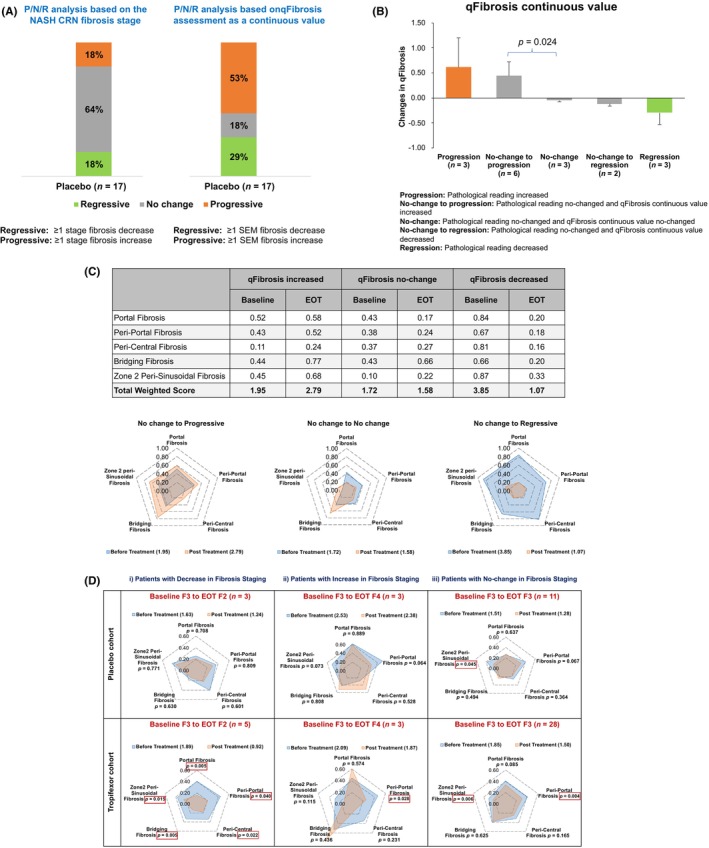
Digital fibrosis quantitation (qFibrosis) in MASH patients with F3 stage liver fibrosis, according to the NASH CRN. (A) Progressive/No Change/Regressive (P/N/R) analysis comparing fibrosis changes as assessed by SHG digital fibrosis quantitation (qFibrosis) versus conventional fibrosis staging from BL to week 48 in patients receiving placebo. (B) qFibrosis changes in subpopulations of MASH F3 fibrosis stage at BL—17 patients receiving placebo. (C) qFibrosis readouts in five areas of the liver lobule presented as a radar map provide graphical view of zonal fibrosis changes from BL to EOT—examples of 3 representative cases categorised as unchanged F3 stage (NASH CRN) at BL and EOT liver biopsies. (D) qFibrosis assessment of fibrosis changes (BL to EOT) presented as a radar map in patients who received placebo or tropifexor. Patients are divided in three subgroups—decreased, increased or no change in fibrosis staging, based on the NASH CRN scoring. The radar maps indicate the mean value of % fibrosis area in different regions of the liver lobules; *p*‐values were calculated by the paired *t*‐test. BL, baseline; CRN, clinical research network; EOT, end of treatment; MASH, metabolic dysfunction‐associated steatohepatitis; NASH, non‐alcoholic steatohepatitis; P/N/R, progressive/no change/regressive; SEM, standard error of the mean; SHG, second harmonic generation.

The P/N/R analysis of fibrosis changes from BL to EOT in patients receiving a placebo (i.e. untreated), based on the NASH CRN scoring showed ‘No Change’ in 64%, with only a small proportion (18%) with fibrosis progression or regression (Figure 1A). In contrast, SHG microscopy with digital quantitation revealed fibrosis progression or regression in 14/17 (82%) of these patients, while only 18% were adjudged as ‘no change’ by this approach. In a further in‐depth analysis, patients were divided into 5 subgroups, taking into account the readouts from both NASH CRN scoring and qFibrosis. The 5 subgroups were (i) fibrosis progression by both assessments; (ii) No change (by NASH CRN) and fibrosis progression (by qFibrosis); (iii) No change by both assessments; (iv) No‐change (by NASH CRH) and fibrosis regression (by qFibrosis) and (v) fibrosis regression by both assessments (Figure 1B). The qFibrosis readout provided greater separation between these five subgroups. There was a significantly greater fibrosis increase in the second subgroup (No change by [NASH CRN] with fibrosis progression [by qFibrosis]) compared to the consensus readout as ‘no change’ by both methods (*p* = .024; Figure 1B).

### 3.2 Radar maps reveal fibrosis dynamics in different regions of liver lobule from BL to EOT

To assess the topography of fibrosis dynamics within the liver lobule, we illustrated qFibrosis changes from BL to EOT using a radar map involving 5 different regions – portal fibrosis, peri‐portal fibrosis, Zone 2 perisinusoidal fibrosis, peri‐central fibrosis, and bridging fibrosis. The radar maps clearly visualised different patterns in fibrosis dynamics in 3 representative cases that were categorised as ‘No Change’ by the NASH CRN scoring, while the qFibrosis result in each of those cases showed either fibrosis progression, no change or regression (Figure 1C). In the case of fibrosis progression, the overall qFibrosis increased from 1.95 (BL) to 2.79 (EOT), while in the case of fibrosis regression, qFibrosis decreased from 3.85 (BL) to 1.07 (EOT), and these dynamics were apparent by differences in the fibrosis areas as shown on the radar maps (Figure 1C).

The radar maps were then applied to analyse separately fibrosis dynamics in 17 patients receiving a placebo and 36 patients receiving TXR (Figure 1D). For this analysis, patients were divided into three subgroups based on fibrosis changes using the NASH CRN scoring – those whose fibrosis increased, decreased or was unchanged from BL to EOT. In the placebo group, the radar map revealed significant fibrosis increase in perisinusoidal fibrosis, while in some patients treated with TXR significant fibrosis reduction was detected in the portal and peri‐portal areas and in bridging fibrosis (Figure 1D).

### 3.3 Comparison of regressive septa versus progressive septa

**FIGURE 2:**
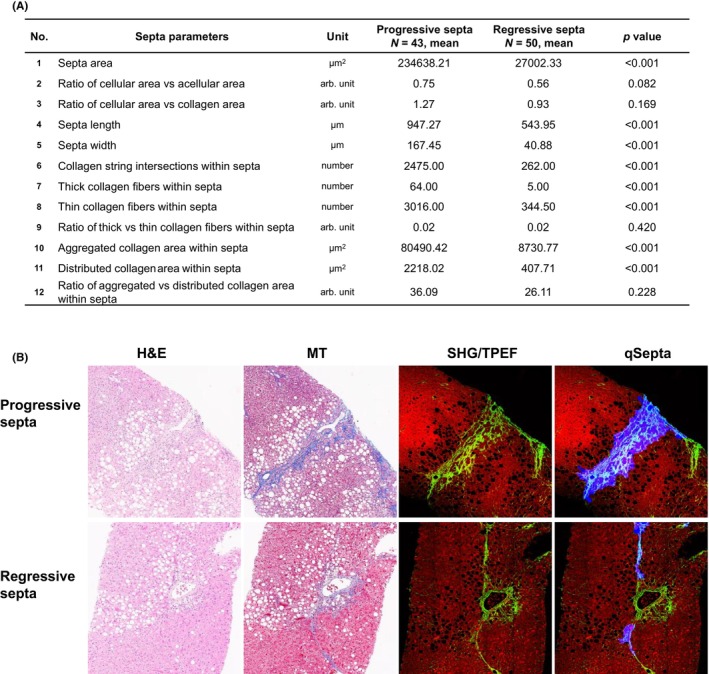
Comparison of progressive and regressive fibrous septa in MASH F3 stage liver fibrosis. (A) The quantitative readouts of 12 individual septa parameters were evaluated in 43 progressive and 50 regressive septa, which were randomly selected from 25 BL liver samples. All septa parameters were normalised by the number of septa in each liver biopsy. The data in the table represent measures of individual septa, and not for the biopsy overall. For example, ‘Septa Area’ refers to the average area of either progressive septa (*n* = 43) or regressive septa (*n* = 50). Septa were defined as ‘Progressive’ or ‘Regressive’ according to the Beijing classification (Sun Y. et al. *Hepatology*. 2017;65:1438–1450). *p*‐values were calculated by the Wilcoxon rank sum test arb. Unit—arbitrary unit. (B) Representative images of Progressive and Regressive septa as visualised with SHG microscopy and with conventional stains. H&E, haematoxylin and eosin; MASH, metabolic dysfunction‐associated steatohepatitis; MT, Masson trichrome; qSepta, fibrous septa defined by the qSepta algorithm are shown in purple highlights; SHG/TPEF, second harmonic generation/two‐photon excitation fluorescence microscopy.

To compare the numerical readouts of 12 individual septa parameters in Progressive and Regressive septa, as previously defined,^20^, ^31^ 93 septa were randomly selected by the AI‐based software from 25 BL liver biopsies, which included 43 progressive and 50 regressive septa (Figure 2A). Out of 12 septa parameters, 8 demonstrated statistically significant differences (*p* < .001) between progressive and regressive septa, that is area, length, width, number of intersections, number of thin and thick fibres, aggregated septa and distributed collagen fibres within septa. These quantitative differences between progressive and regressive septa reflect in detail the visual differences as observed by the conventional stains (H&E and MT) and by SHG microscopy of unstained liver sections (Figure 2B).

### 3.4 Quantitation of treatment‐induced changes in fibrous septa

**FIGURE 3:**
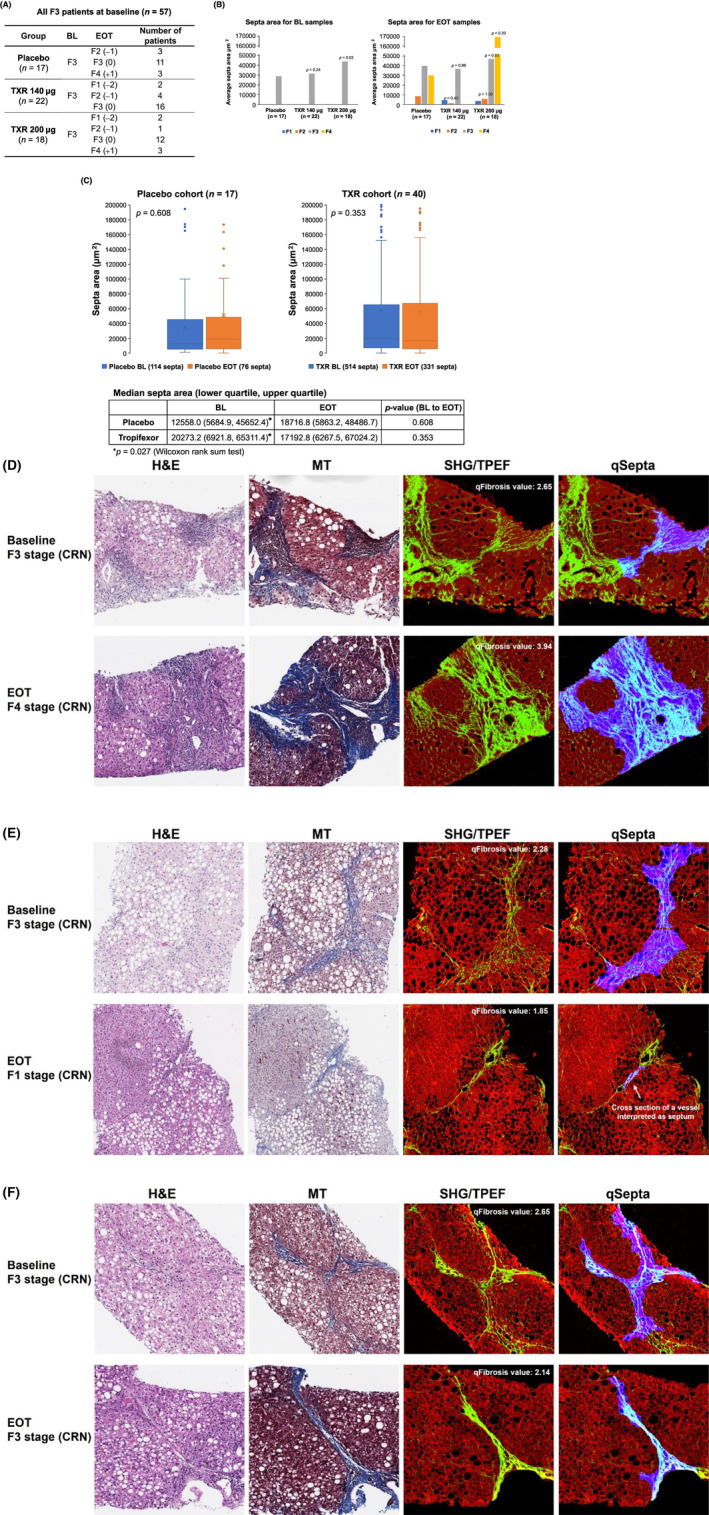
Quantitation of treatment‐induced changes in the septa area and architecture. (A) Liver fibrosis changes (BL to EOT) as assessed by the NASH CRN scoring in all 57 patients, who had MASH F3 stage at BL. (B) Average septa area and septa morphology (according to NASH CRN) in BL and EOT biopsies in patients randomised to receive placebo and two doses—140 or 200 μg tropifexor. The average septa area on the Y‐axis represents the area of all septa normalised by the number of septa in each liver biopsy. The *p*‐values compared to placebo were calculated by Wilcoxon rank sum test. (C) Comparison of the individual septa area in liver biopsies from patients randomised to receive placebo (*n* = 17) or tropifexor (*n* = 40). The data represent the average area of individual septa, and not for the biopsy overall and the boxplot reflects the septa area for every septum at BL or EOT samples. *At BL, the median septa area was significantly higher in the tropifexor (*n* = 40) versus the placebo (*n* = 17) group (*p* = .027). Comparisons were assessed with the Wilcoxon rank sum test. (D–F) Routine stains and SHG/TPEF images of representative cases with fibrosis progression (D), regression (E), and no change in fibrosis stage according to the NASH CRN but with fibrosis regression as assessed by qFibrosis (F). BL, baseline; CRN, clinical research network; EOT, end of treatment; H&E, haematoxylin and eosin; MASH, metabolic dysfunction‐associated steatohepatitis; MT, Masson trichrome; NASH, non‐alcoholic steatohepatitis; P/N/R, progressive/no change/regressive; qSepta, fibrous septa defined by the qSepta algorithm are shown in purple highlights; SHG/TPEF, second harmonic generation/two‐photon excitation fluorescence microscopy; TXR, tropifexor.

Next, we analysed changes in fibrous septa from BL to EOT in patients receiving placebo or TXR. The fibrosis stage was designated as F1 to F4, based on the NASH CRN scoring of pre‐ and post‐treatment liver biopsies (Figure 3A), and the average septa area for the entire liver biopsy was determined by the digital AI software (Figure 3B). At BL, all biopsies were F3 stage; however, the average septa area in patients randomised to receive TXR 200 μg was significantly higher than in the placebo group (*p* = .02, Figure 3B). In the post‐treatment biopsies, the variety of fibrosis stages ranged from F2 to F4 in both placebo and the TXR groups, and there were 4 cases with F1 stage at the EOT in the TXR‐treated patients. In the TXR‐treated patients with EOT fibrosis stage F1 or F2, there were incomplete septa, and the average septa area was markedly reduced from BL; however, the difference was not significant in comparison to the placebo group due to the small number of cases. Note that, in some cases a thin septum detected in early fibrosis stages (F1/F2) could be a cross‐sectional view of a vessel (Figure 3E), which may be interpreted as septum by qFibrosis.

We further analysed the average septa area (i.e. the sum of all septa area/number of septa) for the entire F3 liver biopsy for all 57 samples, as a new cumulative parameter for assessing the severity of bridging fibrosis, and compared the changes from BL to EOT. This analysis revealed, that at BL, the average septa area for the entire liver specimen was significantly higher in patients randomised to TXR than in the placebo group (*p* = .027, Figure 3C). At the EOT, the average septa area in the placebo group was higher than BL, while in TXR‐treated patients the EOT septa area was lower than BL, however, these differences were not significant (*p* = .61 and *p* = .35, respectively; Figure 3C).

Stage 3 fibrosis is generally recognised as very broad and we utilised the advantages of digital AI for a more ‘granular’ characterisation of the F3 stage. For this purpose, all 57 patients were divided into two subgroups based on the characteristics of their septa: (1) F3a—Predominantly regressive septa and (2) F3b—Predominantly progressive septa.

Based on the analysis of septa parameters presented in Figure 2A, a cut‐off for septum width 88.49 μm was used to define regressive (septum width <88.49 μm) and progressive septa (septum width ≥88.49 μm). Considering the common occurrence of both progressive and regressive septa types within a single F3 liver specimen, we quantitatively determined the area of all progressive septa. Patients were then categorised into either the predominantly regressive or predominantly progressive subgroup, based on the ratio of the area of progressive septa to the total septa area within the entire liver specimen.

- Predominantly regressive subgroup (F3a): Area of progressive septa/Area of all septa ratio <50% (range, 0%–48%; mean, 12.9%)
- Predominantly progressive subgroup (F3b): Area of progressive septa/Area of all septa ratio ≥50% (range, 51%–97%; mean, 70.7%)

In the overall group of 57 patients with F3 stage, 27 were categorised as F3a (placebo, *n* = 9; TXR, *n* = 18) and 30 were categorised as F3b (placebo, *n* = 8; TXR *n* = 22). Patients in the 3b subgroup (predominantly progressive) had significantly greater average septa area in the liver biopsy than patients in the 3a subgroup (predominantly regressive)—mean ± SEM, 21820 ± 3383 μm^2^ versus 67 520 ± 7219 μm^2^, respectively (*p* < .001).

Using this more ‘granular’ staging of F3 fibrosis, we analysed the liver fibrosis changes from BL to EOT in patients receiving placebo or TXR (Figure S3).

These quantitative analyses of septa parameters provide a precise characterisation of fibrosis severity and the changes between BL and EOT, which are visualised and subjectively assessed using the conventional microscopy and H&E and MT stains. Representative cases—all with F3 stage at BL but different EOT outcomes—fibrosis progression (Figure 3D); fibrosis regression (Figure 3E), and no change, according to CRN but reduction according to qFibrosis (Figure 3F) illustrate the greater details and precision of digital quantitation in characterising the direction of fibrosis dynamics with progression or regression.

### 3.5 Quantitative analyses of septa changes with radar maps

**FIGURE 4:**
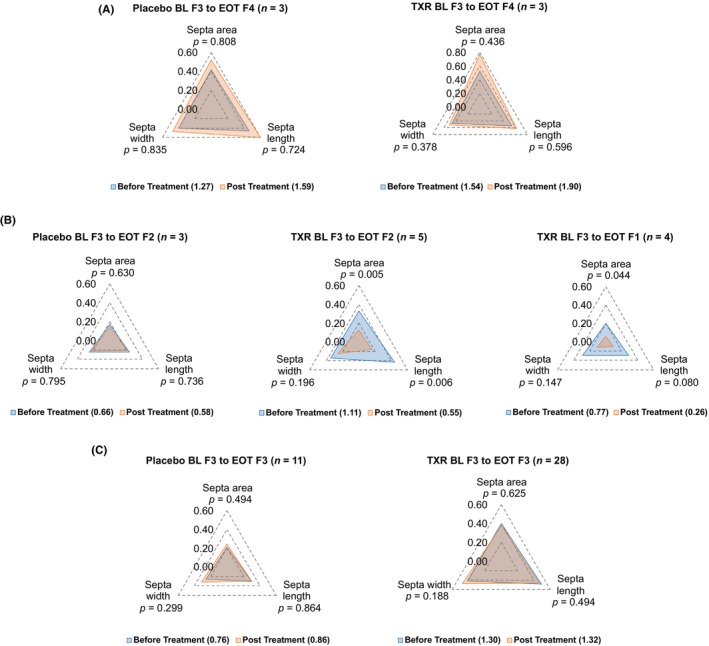
Quantitative analyses of septa changes from BL to EOT with radar maps, illustrating the dynamics in septa area, septa width and septa length in F3 patients with fibrosis progression (A); regression (B), or no change (C) who received placebo or TXR. The septa area, length and width were normalised by the number of septa in the entire liver biopsy. The radar maps indicate the mean value of septa parameters. *p*‐values were calculated by the paired *t*‐test. BL, baseline; CRN, clinical research network; EOT, end of treatment; H&E, haematoxylin and eosin; MASH, metabolic dysfunction‐associated steatohepatitis; MT, Masson trichrome; NASH, non‐alcoholic steatohepatitis; P/N/R, progressive/no change/regressive; qSepta, fibrous septa defined by the qSepta algorithm are shown in purple highlights; SHG/TPEF, second harmonic generation/two‐photon excitation fluorescence microscopy; TXR, tropifexor.

Of the 12 septa parameters, the septa area, length and width were selected for further analyses of the changes (BL to EOT) using the radar maps (Figure 4). In patients with fibrosis progression (from F3 to F4 stage), both in the placebo and in the TXR group, the EOT area on the radar map was clearly larger than the area at BL, however, the changes in the three septa parameters—septa area, length and width did not reach statistical significance, probably due to the small number of cases (*n* = 3) in each subgroup (Figure 4A). In patients with fibrosis regression (from F3 to F2 or F1), the radar map showed marked differences before and at the EOT with a significant reduction in at least one of the septa parameters included in the map (Figure 4B).

### 3.6 Changes in individual septa parameters from BL to EOT in the three subgroups—regression, progression on no change of liver fibrosis

**FIGURE 5:**
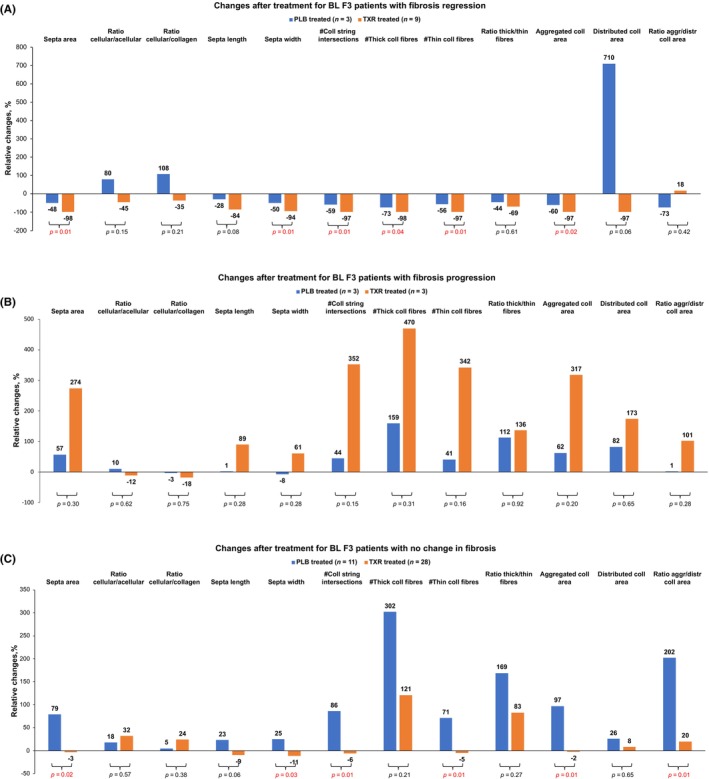
Changes in the individual septa parameters in patients with F3 fibrosis at BL who showed fibrosis regression (A), progression (B), or No Change (C) of liver fibrosis at EOT. The readouts for the septa parameters (except for ratio‐based data) were normalised by the number of septa in the liver biopsy. *p‐*values were calculated by unpaired *t*‐test. The full description for each of the 12 parameters is shown in Figure 2A. aggr, aggregated; BL, baseline; coll, collagen; distr, distributed; EOT, end of treatment; PLB, placebo; TXR, tropifexor.

The changes in each of the 12 septa parameters were next analysed in the subgroups of patients with fibrosis regression, progression or No‐Change at week 48, based on the NASH CRN scoring, both in the placebo and the TXR groups (Figure 3A). Three patients in the placebo group and 9 patients receiving TXR showed >1 stage fibrosis reduction and all 12 septa parameters in the TXR group were reduced more than in the placebo group (Figure 5A). Of note, septa area, width, number of intersections, thick fibres, thin fibres, and aggregated fibres were 6 of the 12 parameters that were reduced significantly more in the TXR group than in the placebo group. There was also greater reduction in the septa length in the TXR group but the statistical significance was borderline (*p* = .08, Figure 5A).

In the subgroup with fibrosis progression at the EOT, there were 3 patients on placebo and 3 who received TXR. Seven of 12 septa parameters showed marked changes (BL to EOT) in both groups, with greater increase in the TXR group, however, the differences were not significant (Figure 5B). In the subgroup of patients categorised as “no change” in fibrosis according to the NASH CRN, 6 of 12 septa parameters were reduced significantly more in the TXR group compared to patients in the placebo group (Figure 5C). The difference in the septa length was again borderline (*p* = .06). Overall, there was consistency in the septa parameters (septa width, area, intersections, number of thin fibres, and aggregated fibres) that were significantly reduced, in the TXR subgroups with regression or no change.

The changes of the 12 septa parameters (BL to EOT) were further analysed by dividing the patients into subgroups, taking into account the readouts from both NASH CRN scoring and digital quantitation (similar to the subgroups as described for Figure 1B). Septa area, length and width again stood out of all 12 and showed statistically significant decrease in patients with fibrosis regression, while several parameters—like septa area, width, intersections, and number of thin or thick fibres showed significantly greater increases in the TXR treated patients compared to those in the placebo group (Figure S4).

### 3.7 qFibrosis model or septa parameters as predictors of outcome—Fibrosis regression versus non‐regression

**FIGURE 6:**
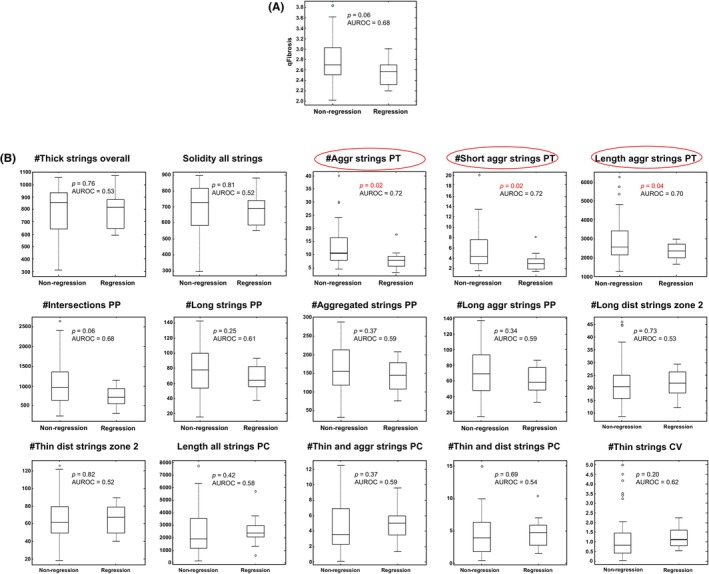
Analysis of BL values of qFibrosis overall and 15 individual qFibrosis parameters as predictors of outcome after 48 weeks in two subgroups of patients with F3 stage at BL: Fibrosis regression versus fibrosis non‐regression. (A) qFibrosis readout as a continuous value. (B) Individual parameters from the qFibrosis model. The full description for each of the 15 parameters is shown in Figure S1. *p*‐values were calculated by unpaired *t*‐test. aggr, aggregated; AUROC, area under the receiver operating characteristic curve; BL, baseline; coll, collagen; CV, central vein; distr, distributed; PC, peri‐central; PP, peri‐portal; PT, portal tract.

We also analysed whether the pre‐treatment readout for the 15 parameters selected from the qFibrosis model or the 12 septa readouts at BL could predict the outcome after 48 weeks with liver fibrosis regression or non‐regression, as shown in Figure 3A. The median of qFibrosis as a continuous value at BL was lower in the subgroup with fibrosis regression versus those with non‐regression (*p* = .06; Figure 6A). Analysis of each of the 15 qFibrosis parameters individually showed that three portal tract (PT) parameters had major contributions for the difference between the two subgroups—the number of aggregated strings in PT; the number of short and aggregated strings in PT and the length of aggregated strings in PT were significantly lower at BL in patients who had fibrosis regression versus those with non‐regression (Figure 6B). Analysis of the individual septa parameters showed that there was no difference between the BL values between the two subgroups—fibrosis regression versus non‐regression (Figure S5).

## 4 DISCUSSION

The present study demonstrates that digital pathology with SHG microscopy and AI analyses provides greater granularity and precision in assessing fibrosis dynamics in MASH patients with bridging fibrosis and reveals worsening or improvement of liver fibrosis below the level of whole‐stage change determined by conventional microscopy. The radar maps of fibrosis dynamics represent a new approach to quantitatively assess and visually present fibrosis dynamics in different areas of the liver lobule and uncover changes with fibrosis progression or fibrosis regression even within the same stage using NASH CRN staging. The in‐depth analysis of 12 individual septa parameters defines quantitative differences between progressive and regressive septa. It identifies new, clinically useful measures, such as the average septa area per liver specimen, to better characterise the status and direction of liver fibrosis evolution. This is evident in the current study population of 57 patients, all with MASH F3 fibrosis stage at BL, where the average septa area revealed a significant imbalance between patients randomised to receive TXR or placebo. These findings reinforce the benefits of using digital pathology with AI in clinical trials with precise quantitation of qFibrosis. The average septa area and other septa parameters could support investigators for better selection and balanced stratification of patients during trial randomisation, as well as for dose–response analyses of antifibrotic agents in the early stages of drug development.

Our findings are concordant with a recent preclinical study showing progressive changes in multiple qFibrosis parameters following a high‐fat sugar‐water diet for 40–52 weeks in DIAMOND mice, as well as fibrosis regression upon diet reversal, even at a point where a full‐stage fibrosis regression was not evident.^30^ The induction of fibrosis progression by exposure to the root cause of the disease, that is, high fat and sugar intake, and regression by removal of the etiological exposures support not only the likelihood that the changes seen in qFibrosis and septa parameters, as determined by SHG microscopy, are reflective of progression and regression but also the interpretation of similar changes in clinical trial settings.^30^ Our earlier findings in patients with MASH suggest that treatment‐induced fibrosis regression starts with a marked reduction in perisinusoidal fibrosis.^13^ There is concordance between this data, our present in‐depth qFibrosis and septa analyses, and the preclinical findings of diet‐induced fibrosis progression and regression in the DIAMOND mouse model. Collectively, these data support the concept that quantitative changes in collagen fibres as assessed by SHG digital pathology reflect early fibrosis regression and could detect and measure such changes before a full‐stage fibrosis improvement is evident via conventional assessment. A recent study has revealed that fibrogenic signalling evolves with liver disease progression, shifting from paracrine signalling to predominantly autocrine hepatic stellate cell (HSC) crosstalk.^32^ Future combination approaches using SHG microscopy, together with spatial transcriptomics, are expected to advance knowledge of fibrogenesis, whether the autocrine signalling circuit would be more active at the bridging fibrosis stage in comparison to established cirrhosis, whether areas in the liver biopsy with more intense fibrosis have more HSC interactions, and whether there are new targets to regulate cell–cell interactions in fibrous bands and reduce autocrine HSC signalling.

Previously, a semiquantitative assessment of liver biopsies from patients with compensated cirrhosis found that fibrous septa thickness is an independent predictor of the development of clinical decompensation.^33^ Thus, precise quantitative assessment of septa parameters in patients with advanced fibrosis can classify patients into subgroups based on objective measures and allow evaluation of their prognostic implications. Importantly, machine‐learning liver histology scores were shown to correlate with the degree of portal hypertension in patients with MASH cirrhosis.^34^, ^35^ This further supports the application of quantitative digital pathology assessment for evaluating changes in liver disease progression and regression.

The development, validation, and standardisation of digital pathology with AI to evaluate liver histology have been recognised as an area of high priority in the MASH field, particularly for drug development.^36^ A recent comprehensive review of this approach has mapped an agenda for future research and development.^37^ The importance of liver fibrosis, and no other histological features, in predicting outcomes in MASLD has been highlighted by several studies.^5^, ^6^, ^7^, ^8^, ^9^, ^10^ Furthermore, in patients with compensated cirrhosis due to MASH, regression of fibrosis is associated with a reduction in liver‐related complications.^38^ Thus, histopathological assessment of liver biopsies is mandatory as a primary endpoint for conditional drug approval; however, this represents one of the main challenges in drug development for MASH because of the limitations of the semiquantitative ordinal scoring systems and the variability in the histological assessment.^37^, ^39^ The present findings extend the growing body of evidence that the standard categorical semiquantitative histologic scoring systems are inadequate for assessing liver fibrosis progression and regression, both in routine clinical practice and in clinical trials, and the need to incorporate digital pathology with AI quantitation in liver biopsy assessment as an aid to pathologists. The use of qFibrosis has been shown to substantially improve the inter‐rater concordance, with a mean linearly weighted Kappa of .82, as compared with .72 for the unassisted review.^40^

The present study has certain limitations. In particular, a modest number of patients (57 cases with MASH F3 fibrosis) and a relatively short duration (48 weeks follow‐up). A longer follow‐up and possibly a third liver biopsy would have revealed greater differences between the parameters measured and their significance. In the future, purposefully designed studies should evaluate the reliability of different fibrosis parameters and the potential impact of sample size variation. The clinical relevance of qFibrosis changes, along with the individual septa parameters, will need to be established in longitudinal studies. The first evidence that SHG‐derived digital pathology readouts allow direct prediction of all‐cause mortality, hepatic decompensation, and hepatocellular carcinoma development in patients with MASLD was presented this year at the International Liver Congress.^41^

In summary, the present study provides new evidence that digital pathology with SHG microscopy and AI analyses allows precise characterisation of fibrosis severity and changes. These changes, with fibrosis progression or regression, may be part of the natural evolution of the disease, or treatment‐induced and are not detected by conventional liver histology assessment. The fibrosis parameters evaluated in this study—qFibrosis overall and different areas in liver lobules, as well as individual septa parameters—apply to liver fibrosis of any aetiology and demonstrate the overarching applicability of this approach. Wider clinical application of this more sensitive and detailed assessment of fibrosis changes will allow pathologists to provide more granular information in routine clinical practice. It will also improve the evaluation of therapeutic interventions in clinical trials for MASH and other liver diseases as well as liver research for a better understanding of fibrosis biology and evolution. Patients with bridging fibrosis, especially with high disease activity, are most likely to progress to cirrhosis.^18^ Key elements in the future MASLD management will be to reduce fibrogenesis in this population, prevent progression to cirrhosis, and assess the risks of portal hypertension and hepatocellular carcinoma development.^42^ Digital pathology will be an invaluable tool in these efforts.

## AUTHOR CONTRIBUTIONS

Conceptualisation and study design: NVN, DEK, DB, CS, DT, AJS; Second Harmonic Generation microscopy: EC, YR, DT; Data collection and analyses: NVN, EC, YR, DEK, DT, AJS; Drafting of manuscript: NVN, EC; All authors contributed to the discussions and interpretation of study results; All authors contributed to the manuscript preparation and approved the final version of the article.

## FUNDING INFORMATION

This study was supported by Novartis Global Drug Development, Basel, Switzerland and HistoIndex Pte. Ltd, Singapore. This study was supported in part by the Intramural Research Program of the National Institutes of Health, National Cancer Institute.

## CONFLICT OF INTEREST STATEMENT

NV Naoumov: Advisor, HistoIndex; DE Kleiner: Uncompensated collaborations with HistoIndex and HighTide.; D. Brees and C. Saravanan: Employee, Novartis; E Chng, Y Ren and D Tai: Employee, Histoindex. AJ Sanyal: Stock options in Genfit, Akarna, Tiziana, Indalo, Durect Inversago, and Galmed. He has served as a consultant to Astra Zeneca, Nitto Denko, Conatus, Nimbus, Salix, Tobira, Takeda, Jannsen, Gilead, Terns, Birdrock, Merck, Valeant, Boehringer Ingelheim, Bristol Myers Squibb, Lilly, Hemoshear, Zafgen, Novartis, Novo Nordisk, Pfizer, Exhalenz, and Genfit. He has been an unpaid consultant to Intercept, Echosens, Immuron, Galectin, Fractyl, Target‐Pharma, Syntlogic, Affimune, Chemomab, Zydus, Nordic Bioscience, Albireo, Prosciento, and Surrozen. His institution has received grant support from Gilead, Salix, Tobira, Bristol Myers, Shire, Intercept, Merck, Astra Zeneca, Malinckrodt, Cumberland, and Novartis. He receives royalties from Elsevier and UptoDate.

## Supporting information


Figure S1:

Figure S2:

Figure S3:

Figure S4:

Figure S5:

Data S1:

## Data Availability

The data that support the findings of this study are included within the article and its supplementary materials. Data can be made available for collaborative investigations upon request with an appropriate institutional collaboration agreement.
