# Supplementary material for: Digital quantitation of bridging fibrosis and septa reveals changes in natural history and treatment not seen with conventional histology
Source: Liver Int. 2024 Sep 9;44(12):3214–28. doi: 10.1111/liv.16092 (PMC11586893; doi:10.1111/liv.16092)
Supplement: Supplementary file 1 — Figure S1: [file LIV-44-3214-s001.pdf]

No.	Parameters	Region
1	Number of thick strings for overall fibrosis	Overall
2	Solidity of all strings for overall fibrosis	
3	Number of aggregated strings for portal tract fibrosis	Portal tract
4	Number of short and aggregated strings for portal tract fibrosis	
5	Length of aggregated strings for portal tract fibrosis	
6	Number of intersections of all strings for peri-portal fibrosis	Peri-portal
7	Number of long strings for peri-portal fibrosis	
8	Number of aggregated strings for peri-portal fibrosis	
9	Number of long and aggregated strings for peri-portal fibrosis	
10	Number of long and distributed strings for zone 2 fibrosis	Zone 2
11	Number of thin and distributed strings for zone 2 fibrosis	
12	Length of all strings for peri-central fibrosis	Peri-central
13	Number of thin and aggregated strings for peri-central fibrosis	
14	Number of thin and distributed strings for peri-central fibrosis	
15	Number of thin strings for central vein fibrosis	Central vein
