# Supplementary material for: Digital quantitation of bridging fibrosis and septa reveals changes in natural history and treatment not seen with conventional histology
Source: Liver Int. 2024 Sep 9;44(12):3214–28. doi: 10.1111/liv.16092 (PMC11586893; doi:10.1111/liv.16092)
Supplement: Supplementary file 2 — Figure S2: [file LIV-44-3214-s005.pdf]

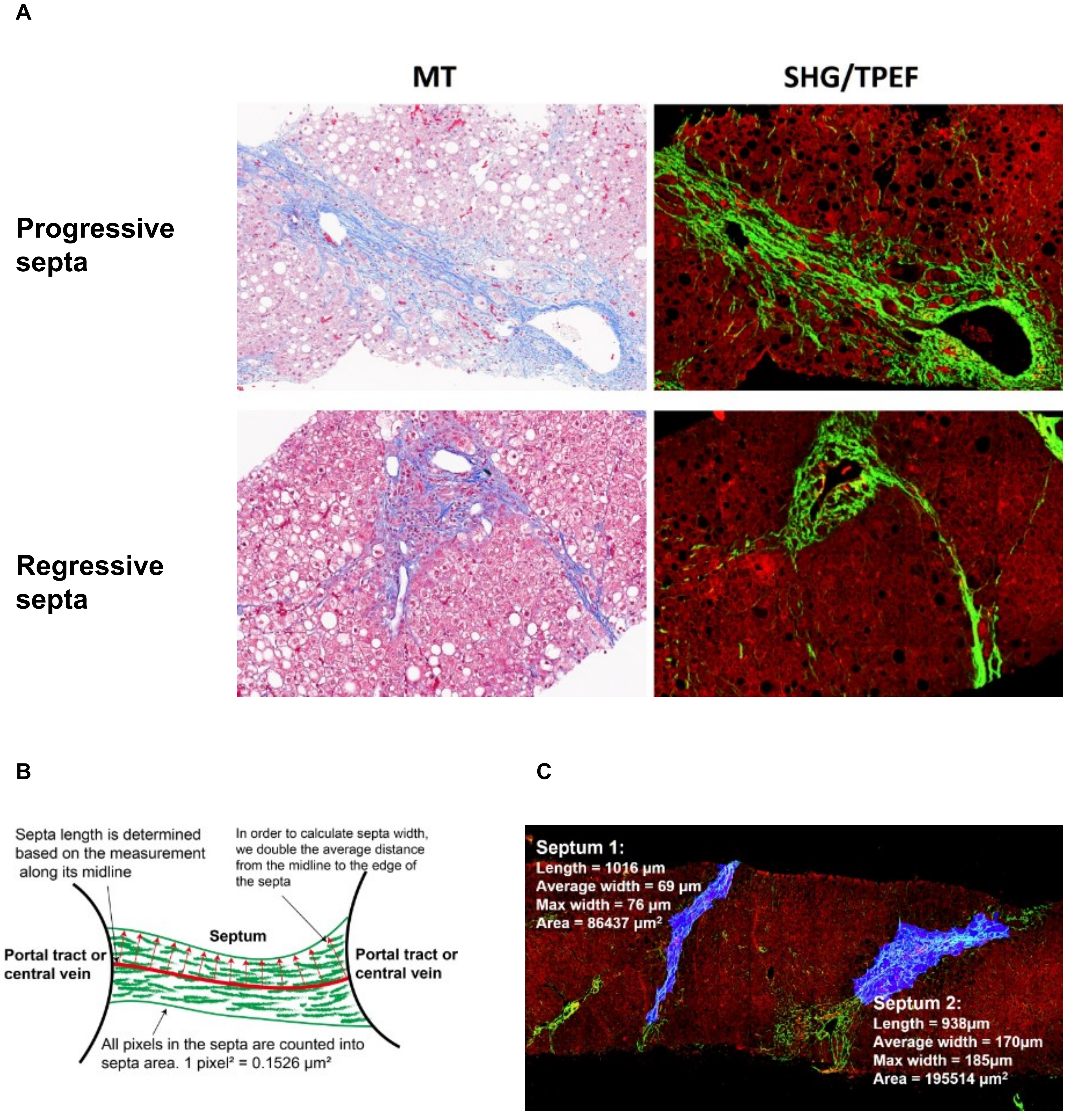
A
MT
SHG/TPEF
Progressive septa
Regressive septa
B
Septa length is determined based on the measurement along its midline
In order to calculate septa width, we double the average distance from the midline to the edge of the septa
Septum
Portal tract or central vein
Portal tract or central vein
All pixels in the septa are counted into septa area. 1 pixel² = 0.1526 µm²
C
Septum 1:
Length = 1016 µm
Average width = 69 µm
Max width = 76 µm
Area = 86437 µm²
Septum 2:
Length = 938µm
Average width = 170µm
Max width = 185µm
Area = 195514 µm²
