# Supplementary material for: Digital quantitation of bridging fibrosis and septa reveals changes in natural history and treatment not seen with conventional histology
Source: Liver Int. 2024 Sep 9;44(12):3214–28. doi: 10.1111/liv.16092 (PMC11586893; doi:10.1111/liv.16092)
Supplement: Supplementary file 3 — Figure S3: [file LIV-44-3214-s003.pdf]

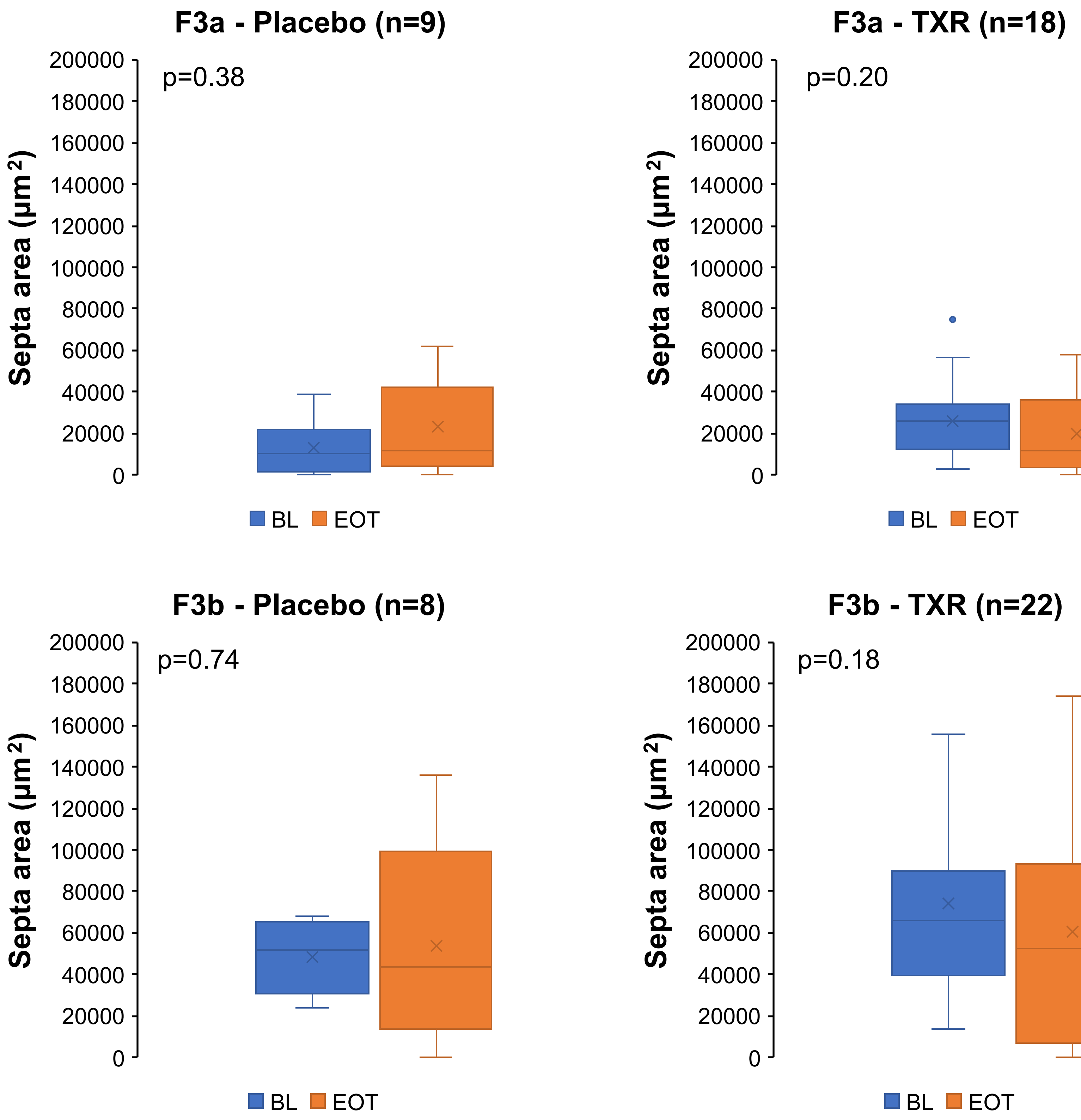


**Median septa area (lower quartile, upper quartile), μm$^2$**

| | | BL | EOT | p-value (BL to EOT) |
|---|---|---|---|---|
| **F3a** | **Placebo** | 10524.4 (1329.4, 22015.5) | 11474.6 (4123.7, 42553.8) | 0.38 |
| | **Tropifexor** | 26046.6 (12326.3, 34220.4) | 11977.5 (3619.2, 36068.4) | 0.20 |
| **F3b** | **Placebo** | 51999.7 (30762.6, 65272.9) | 43822.3 (13633.6, 99577.3) | 0.74 |
| | **Tropifexor** | 66243.9 (39611.6, 90085.4) | 52330.7 (6867.6, 93468.4) | 0.78 |
