# Supplementary material for: Digital quantitation of bridging fibrosis and septa reveals changes in natural history and treatment not seen with conventional histology
Source: Liver Int. 2024 Sep 9;44(12):3214–28. doi: 10.1111/liv.16092 (PMC11586893; doi:10.1111/liv.16092)
Supplement: Supplementary file 4 — Figure S4: [file LIV-44-3214-s004.pdf]

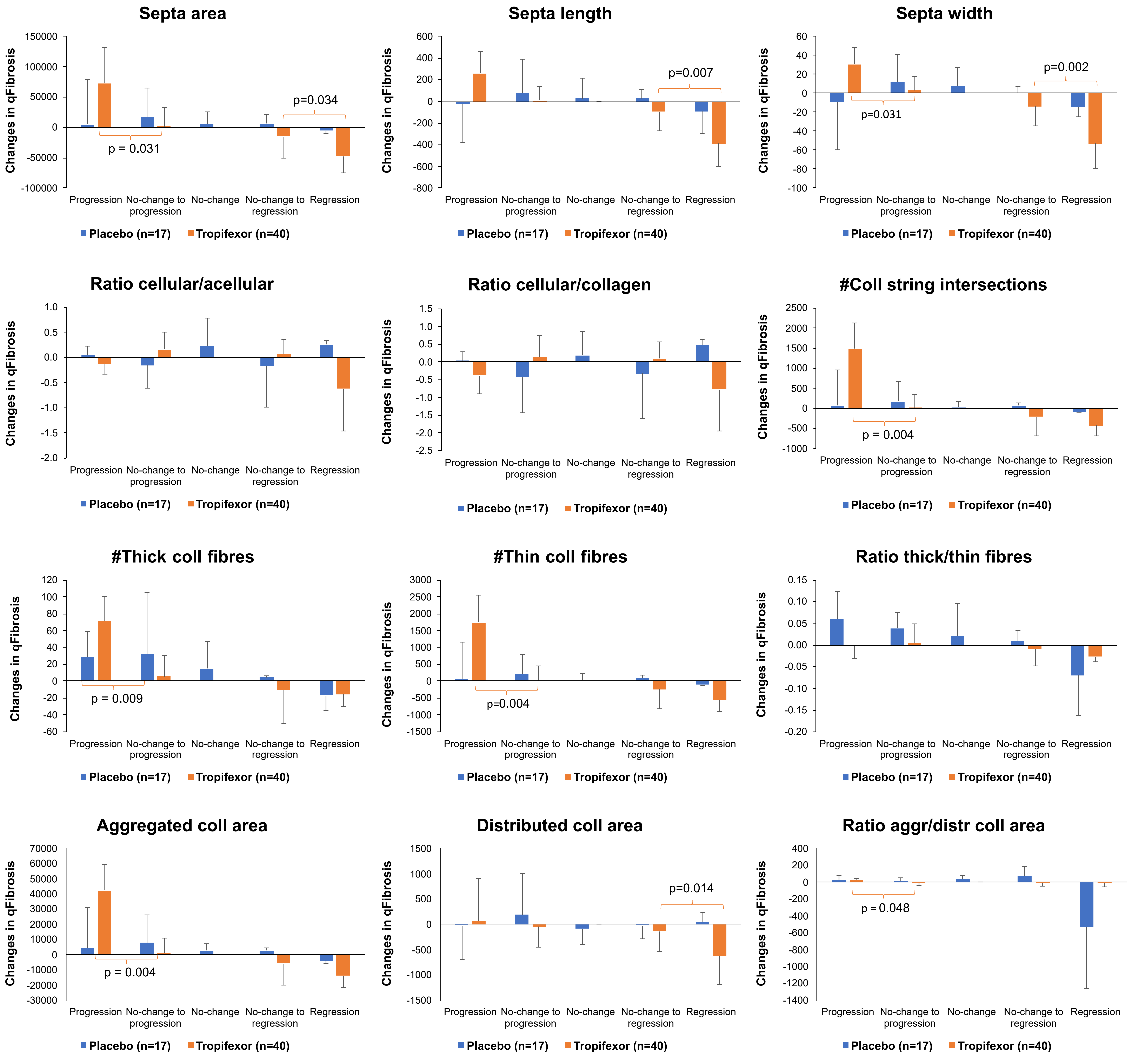

Progression: Pathological reading increased (Placebo, n=3; Tropofexor, n=3)
No-change to progression: Pathological reading no-changed and qFibrosis continuous value increased (Placebo, n=6; Tropofexor, n=12)
No-change: Pathological reading no-changed and qFibrosis continuous value no-changed (Placebo, n=3; Tropofexor, n=0)
No-change to regression: Pathological reading no-changed and qFibrosis continuous value decreased (Placebo, n=2;Tropofexor, n=16)
Regression: Pathological reading decreased (Placebo, n=3;Tropofexor, n=9)
