# Supplementary figures and images for: Digital quantitation of bridging fibrosis and septa reveals changes in natural history and treatment not seen with conventional histology
Source: Liver Int. 2024 Sep 9;44(12):3214–28. doi: 10.1111/liv.16092 (PMC11586893; doi:10.1111/liv.16092)

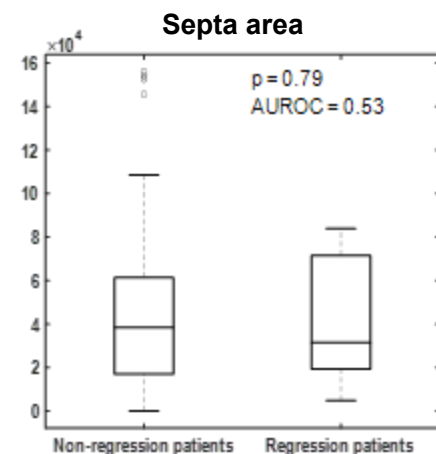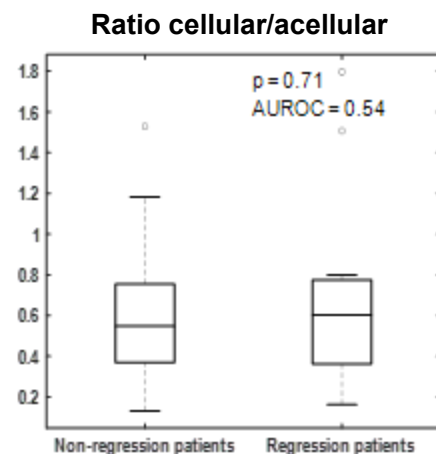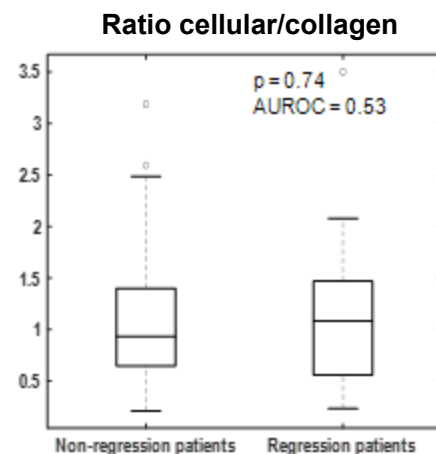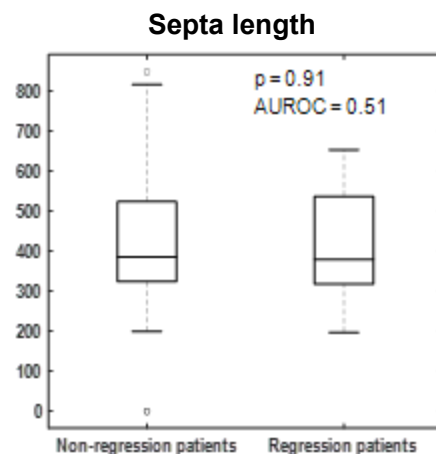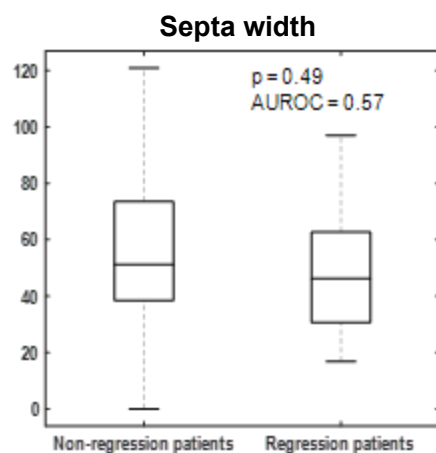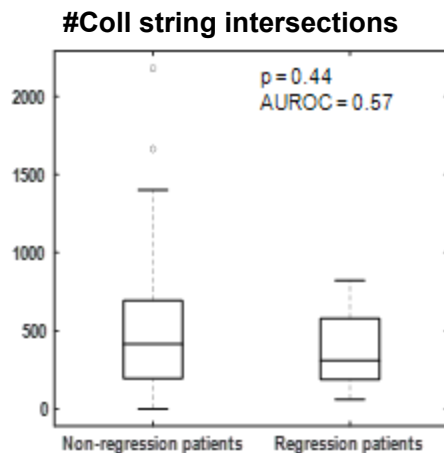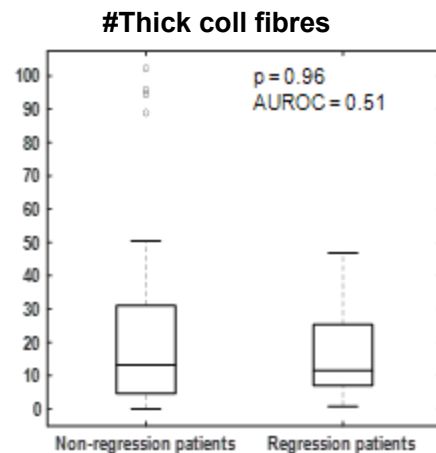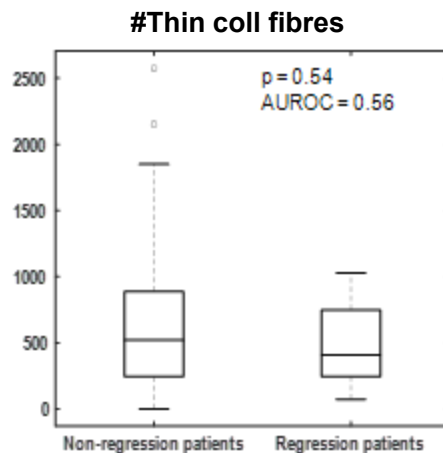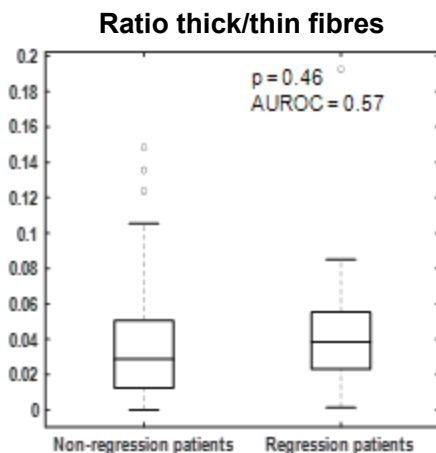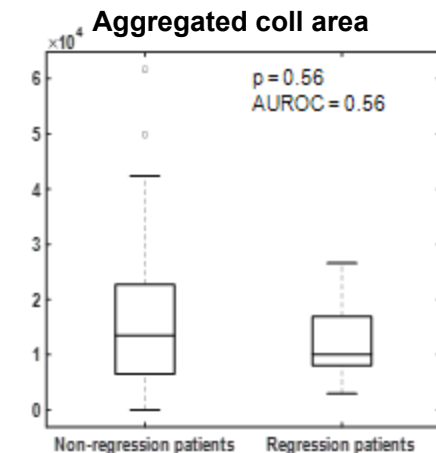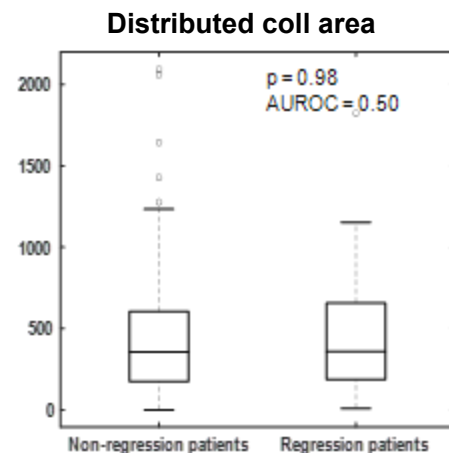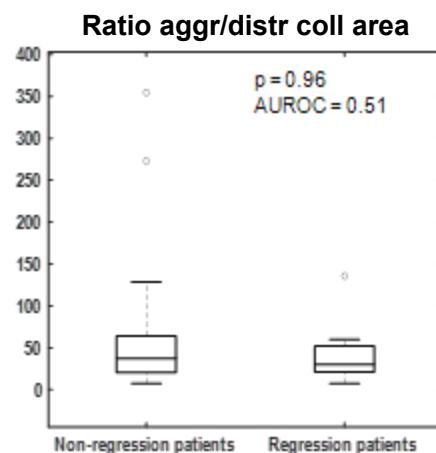

Supplement: Supplementary file 5 — Figure S5: [file LIV-44-3214-s002.pdf]
