# Supplementary material for: Digital quantitation of bridging fibrosis and septa reveals changes in natural history and treatment not seen with conventional histology
Source: Liver Int. 2024 Sep 9;44(12):3214–28. doi: 10.1111/liv.16092 (PMC11586893; doi:10.1111/liv.16092)
Supplement: Supplementary file 6 — Data S1: [file LIV-44-3214-s006.docx]

**SUPPLEMENTARY MATERIAL**

**Supplementary Figure 1:** List of fifteen parameters from the qFibrosis model that were selected and analysed individually.

**Supplementary Figure 2:** Fibrous septa analyses. A) Images of parallel sections from the same liver specimen with progressive or regressive septa visualized with the MT stain and SHG microscopy. B) Schematic illustration of measuring the length, width and area of an individual septum; C) Examples of two septa with measurements of their length, average and maximum width and septa area.

MT, Mason trichrome; SHG/TPEF, second harmonic generation/two-photon excitation fluorescence microscopy.

**Supplementary Figure 3:** Changes of the average septa area in the two subpopulations – F3a and F3b, of patients with MASH F3 fibrosis from BL to EOT. The septa area on the Y-axis represents the area of all septa normalized by the number of septa in each liver biopsy.

P-values were calculated by the Wilcoxon signed rank test.

BL, baseline; EOT, end of treatment; MASH, metabolic dysfunction-associated steatohepatitis; PLB, placebo; TXR, tropifexor.

**Supplementary Figure 4:** Changes of individual septa parameters in the subpopulations of patients as identified by the PNR analysis of fibrosis changes from BL to EOT and comparison of patients receiving TXR (n=40) vs PLB (n=17). The readouts for the septa parameters (except for ratio-based data) were normalized by the number of septa in the liver biopsy. P-values were calculated by the paired t-test. The full description for each of the 12 parameters is shown in Figure 2A. aggr, aggregated; BL, baseline; coll, collagen; distr, distributed; EOT, end of treatment; MASH, metabolic dysfunction-associated steatohepatitis; PLB, placebo; PNR, progressive/no change/regressive; TXR, tropifexor.

**Supplementary Figure 5:** Analysis of the baseline values of 12 septa parameters as predictors of outcome after 48 weeks in two subgroups of patients with F3 stage at baseline - fibrosis regression vs fibrosis non-regression. The readouts for the septa parameters (except for ratio-based data) were normalized by the number of septa in the liver biopsy.

*P-*values were calculated by unpaired t-test. The full description for each of the 12 parameters is shown in Figure 2A.

aggr, aggregated; AUROC, area under the receiver operating characteristic curve; coll, collagen; distr, distributed.
